# The full health, economic, and social benefits of prospective Strep A vaccination

**DOI:** 10.1038/s41541-023-00758-z

**Published:** 2023-10-30

**Authors:** Daniel Cadarette, Maddalena Ferranna, Jeffrey W. Cannon, Kaja Abbas, Fiona Giannini, Leo Zucker, David E. Bloom

**Affiliations:** 1 Harvard Kennedy School, Cambridge, MA USA; 2 University of Southern California Alfred E. Mann School of Pharmacy and Pharmaceutical Sciences, Los Angeles, CA USA; 3 Wesfarmers Centre for Vaccines and Infectious Diseases, Telethon Kids Institute, University of Western Australia, Perth, Australia; 4 London School of Hygiene & Tropical Medicine, London, United Kingdom; 5 School of Tropical Medicine and Global Health, Nagasaki University, Nagasaki, Japan; 6 Harvard T.H. Chan School of Public Health, Boston, MA USA

**Keywords:** Public health, Bacterial infection

## Abstract

Recent research has documented a wide range of health, economic, and social benefits conferred by vaccination, beyond the direct reductions in morbidity, mortality, and future healthcare costs traditionally captured in economic evaluations. In this paper, we describe the societal benefits that would likely stem from widespread administration of safe and effective vaccines against *Streptococcus pyogenes* (Strep A), which was estimated to be the fifth-leading cause of infectious disease deaths globally prior to the COVID-19 pandemic. We then estimate the global societal gains from prospective Strep A vaccination through a value-per-statistical-life approach. Estimated aggregate lifetime benefits for 30 global birth cohorts range from $1.7 to $5.1 trillion, depending on the age at which vaccination is administered and other factors. These results suggest that the benefits of Strep A vaccination would be large and justify substantial investment in the vaccines’ development, manufacture, and delivery.

## Introduction

*Streptococcus pyogenes* (Strep A) causes a wide range of clinical endpoints, ranging from superficial infections of the throat and skin to severe autoimmune diseases (e.g., acute rheumatic fever) and their chronic sequelae (e.g., rheumatic heart disease [RHD]). Strep A was estimated to be the fifth-leading cause of infectious disease deaths among all pathogens prior to the COVID-19 pandemic^1–3^. Effective treatment for Strep A exists in the form of antibiotics, such as penicillin. However, the potential for existing countermeasures to remedy the unaddressed health burden of Strep A is limited by several factors. These include insufficient access to antibiotics in low-resource settings where populations are particularly vulnerable to Strep A; the need for repeated treatment where Strep A is endemic; growing concerns over the development of antibiotic resistance from widespread antibiotic consumption, particularly in bystander pathogens; and the potential for dysbiosis arising from antibiotic treatment to contribute to chronic health problems.

Recognizing the significance of the global disease burden imposed by Strep A and the insufficiency of existing countermeasures, the World Health Organization (WHO) developed a “Group A *Streptococcus* Vaccine Development Technology Roadmap” and preferred product characteristics for Strep A vaccines in 2018^4,5^. As of February 2023, eight Strep A vaccine candidates had shown promise in early development, with the most advanced candidate having successfully completed a Phase 1a clinical trial^6^. The existence of natural immunity to Strep A offers further evidence of the scientific feasibility of vaccine development^7^, and recent safety evaluations of vaccine candidates in humans and animals have helped allay concerns that Strep A vaccination could prompt an adverse autoimmune response^8,9^. Given the scientific feasibility of Strep A vaccine development, widespread vaccination against Strep A is an attractive prospect.

Mounting evidence indicates that vaccination yields broad health, economic, and social benefits, well beyond healthcare cost savings and reductions in deaths and cases of illness^10–15^. In the case of prospective Strep A vaccination, these broad benefits are likely to include, for instance, mitigation of antimicrobial resistance and improvements in educational attainment and labor force participation. In this paper, we estimate the magnitude of Strep A vaccination’s global societal benefits through a willingness-to-pay approach. These estimates build on results from a static cohort model of Strep A vaccination’s projected impact on global disease burden^16^. The model projects country-specific numbers of episodes of clinical disease, deaths, and disability-adjusted life years (DALYs) averted over time under different Strep A vaccination scenarios.

To then project the magnitude of broad benefits from prospective Strep A vaccination, we rely on established estimates of the value of averting one lost year of life, known as the value-per-statistical life year (VSLY). Conceptually, these VSLY estimates capture both the intrinsic and instrumental values of health improvements, i.e., the value of continuing to experience the joys of life itself for a longer period and the value of any changes in wellbeing attributes (e.g., income or medical costs) associated with the risk reductions. VSLY therefore is in principle able to capture individual-level broad benefits of vaccination (e.g., protection against dysbiosis, increased earnings, and improved quality of life). It also captures any spillover effects that have been internalized by individuals. For example, vaccinated individuals may recognize and value the fact that family members will benefit from their vaccination through reductions in caregiving costs or alleviation of mental health burden.

It is well established that VSLY estimates depend on individuals’ ability to pay and thus increase with income. To avoid ethically problematic undervaluation of the benefits of Strep A vaccination experienced by lower-income countries^17^, we adopt a single global estimate of VSLY to be applied to all countries when assessing the global benefits of Strep A vaccination. A comprehensive description of the methodology is included at the end of the paper. This study followed the Consolidated Health Economic Evaluation Reporting Standards (CHEERS) guidelines.

We estimate that lifetime benefits for 30 global birth cohorts are on average $2.3 trillion (for vaccination at birth) and $3.8 trillion (for childhood vaccination). Understanding the magnitude of societal benefits Strep A vaccination can be anticipated to produce is useful for guiding societal investment decisions regarding the development, manufacture, and delivery of safe and effective Strep A vaccines. For example, estimates of the magnitude of societal benefits from Strep A vaccination may inform governmental decisions concerning the allocation of limited resources among various health and non-health interventions (e.g., development of Strep A vaccines versus investment in infrastructure projects), or it may inform the spending strategies of international donors and non-profit organizations interested in reducing the burden of Strep A or improving global health generally. The societal perspective adopted in this paper is complementary to recent research investigating the value of prospective Strep A vaccination from a health sector perspective (health-centric cost-effectiveness analysis)^18^ and a commercial perspective (return on investment for vaccine developers)^6^. In both cases, Strep A vaccination has been found to be a favorable target for investment under most conditions. Additionally, a previous study focused on the Australian context found that a Strep A vaccine that prevented both throat and skin infections would have a cost-effectiveness price similar to the prices of other publicly funded vaccinations^19^.

Here, we describe some of Strep A vaccination’s anticipated health, economic, and social benefits based on literature documenting negative impacts of Strep A diseases and the positive impacts of existing vaccines against other infectious diseases. Table 1 summarizes these benefits and their distribution across different social strata.

**Table 1:** Health, economic, and social benefits of prospective Strep A vaccination and their distribution.

	Vaccination benefits	Individual	Family/ household	Health sector	Society
Health benefits	Direct health effects • Reduced morbidity & mortality directly due to Strep A diseases • Adverse effects of vaccination (negative benefit)	✓			
	Prevention of secondary individual (physical) health effects • Aggravation of comorbidities • Nosocomial infections • Microbiome disruption	✓			
	Mitigation of secondary population-level health effects • Disease transmission • Antimicrobial resistance • Healthcare congestion			✓	✓
	Improved mental health	✓	✓		
Economic benefits	Reduced healthcare costs	✓	✓	✓	
	Reduced caregiving costs	✓	✓	✓	
	Reduced transportation costs	✓	✓		
	Increased labor force participation, hours worked, and income	✓	✓		✓
	Increased productive non-market activities	✓	✓		✓
	Improved educational attainment, school attendance, and cognition	✓			✓
	Fiscal impacts • Increased tax receipts • Reduced public health spending			✓	✓
	Increased wealth/savings	✓	✓		
	Reduced risk and severity of impoverishment	✓	✓		✓
	Reduced risk of economically disruptive outbreaks			✓	✓
Social benefits	Improved social equity				✓
	Intergenerational benefits		✓		
	General risk reduction	✓	✓	✓	✓
	Improved quality of life	✓	✓		✓
	Reduced stigma	✓	✓	✓	✓

Beyond direct prevention of morbidity and mortality from Strep A diseases^16^, potential major health benefits of Strep A vaccination include its anticipated ability to reduce antibiotic consumption and, potentially, antimicrobial resistance (AMR). High amounts of antibiotics are consumed globally to treat Strep A diseases, in particular Strep A pharyngitis^20,21^. Although no significant resistance to penicillin (the first-line antibiotic class of choice for treating superficial infections) has been detected in Strep A, Strep A resistance to other antibiotics that are sometimes used as treatments (e.g., erythromycin) has been detected^22,23^. In addition, rates of penicillin consumption appear to be positively correlated with levels of penicillin resistance in other high-burden pathogens, such as *Streptococcus pneumoniae*^24^. Reducing the incidence of Strep A pharyngitis through vaccination could have a major impact on both necessary and unnecessary antibiotic consumption. One recent study estimated that Strep A vaccination broadly administered to children could conservatively reduce antibiotic consumption for Strep A pharyngitis among children aged 5–14 years by 32% and among all ages by 7%^21^. Moreover, a modeling study of the global and regional burdens of bacterial AMR avertable by a hypothetical Strep A vaccine providing five years of protection at 70% efficacy and 70% coverage and administered at six weeks of age found that vaccination could have averted around 800 deaths and 69,000 DALYs associated with bacterial AMR globally in 2019^25^.

Another health benefit of note is Strep A vaccination’s potential to prevent microbiome disruption from consumption of antimicrobials, which can lead to future infections or contribute to chronic ill health at the individual level^26^. For both vaccinated individuals and their families/households, the health benefits of Strep A vaccination may also include avoidance of significant mental health burdens associated with severe physical disease^27^.

Beyond direct healthcare cost savings stemming from reduced need for treatment, broader economic benefits of vaccination are often ignored in economic analyses. In particular, Strep A vaccination is likely to have an outsized positive impact on educational attainment, school attendance, and cognitive function—all of which have been demonstrated for other vaccines^12^—due to the disproportionate burden the pathogen places on children. The high incidence of pharyngitis and impetigo in children (coupled with the transmissible nature of Step A) leads to frequent absences among schoolchildren^28^. Given the relatively early age of onset for severe manifestations, such as RHD, more significant educational disruptions related to ongoing health impacts and disease management are also possible. Indeed, guidelines recommend that children with a history of acute rheumatic fever or RHD are provided monthly intramuscular injections of penicillin to prevent further Strep A infections and worsening of disease^29^. In more severe cases, frequent specialist (e.g., cardiologist) follow-up is recommended and may necessitate burdensome travel to major cities in settings with limited healthcare resources^30^. Additionally, Strep A vaccination could substantially reduce the risk of economically disruptive school-based outbreaks^31^.

In adults, Strep A can diminish labor force participation, productivity, and income. This is true both for adults directly affected by RHD and other severe Strep A diseases, who may suffer from physical limitations, and for adults who serve as caretakers of children suffering from illness. In addition, premature mortality from Strep A diseases removes individuals from participation in the labor force. Estimated average productivity loss due to premature mortality per episode of RHD ranges from $9637 in low-income countries to $72,097 in high-income countries^32^.

Finally, Strep A vaccination is likely to have several positive social effects. Prevention of Strep A diseases could lead to substantial improvements in social equity both across and within populations. That is because the global distribution of Strep A’s health and economic burdens falls disproportionately on low- and middle-income countries^3,33^, and within countries the burden typically falls disproportionately on low-income and otherwise disadvantaged groups, such as indigenous communities in Australia and New Zealand^34,35^. In practice, any equity improvements a Strep A vaccine may promise are contingent on widespread access that is not predicated on ability to pay for vaccination.

Additional social benefits of vaccination may include better quality of life—beyond improved health status—for individuals who would otherwise suffer long-term effects from Strep A diseases. As one concrete example, women suffering from RHD are sometimes discouraged by physicians from having children due to their disease status^36^. Social benefits of vaccination may also include reduced stigma among RHD patients. For example, a mixed methods study of 75 women living with RHD in Uganda found that more than a quarter of participants in one focus group had been left by their partners due to perceived fertility limitations, and another third of participants feared such abandonment^36^.

## Results

### Evaluation method

Health-centric cost-effectiveness analysis typically focuses narrowly on direct health benefits and on cost savings in the healthcare system, thereby neglecting many of the broader health, economic, and social benefits discussed above^14^. To quantitatively value (at least part of) the broad societal benefits of prospective Strep A vaccination, we rely on established value-per-statistical-life-year estimates. VSLY measures the monetary value of averting a year of life lost. In the absence of specific estimates for Strep A, we follow standard guidelines for the conduct of benefit-cost analysis^37^ and assume that VSLY is proportional to per-capita income^38,39^. Following the relevant literature^40^, we assume that VSLY also measures the monetary value of averting a year of life with disability. Future monetary benefits are discounted at a positive yearly rate. In this analysis, the broad benefits of Strep A vaccination are defined as the present discounted value of future monetary benefits associated with the number of years of life lost and years of life with disability averted across countries and over time thanks to vaccination (see the Methods section for more details).

Estimates of the health benefits of Strep A vaccination are derived from an epidemiological static cohort model developed by Giannini et al. (2023)^16^. The model projects the country-specific number of episodes or cases of clinical disease, deaths, and DALYs averted over time under six different Strep A vaccination scenarios. The scenarios differ in terms of vaccination coverage, year of vaccine introduction, and length of vaccine effectiveness. In all six scenarios, we compute the lifetime benefits of vaccination for 30 vaccinated cohorts, from 2022 to 2051. We also compare a vaccine that is administered at birth with a vaccine that is administered at age five (details about the model are provided in the Methods section).

**Fig. 1:**
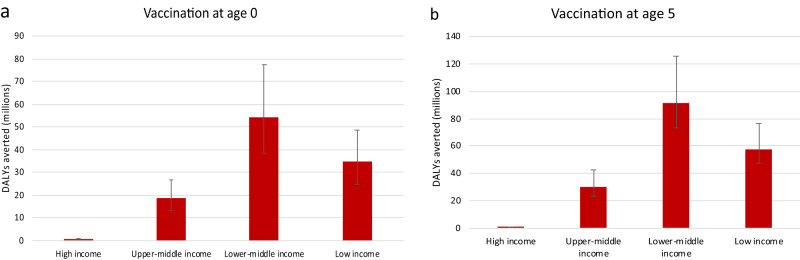
Average number of DALYs averted (in millions) across the six vaccination scenarios by income group. Panel **a** shows the average number of DALYs averted when vaccination occurs at age 0, and Panel **b** shows the average number of DALYs averted when vaccination occurs at age 5. We assume zero discounting to compute the total number of DALYs averted per scenario. The colored bars represent the average number of DALYs averted across the six vaccination scenarios, while the black bars denote the variation across scenarios. Please note that the scales of the y-axes differ between the two panels.

Overall, the model predicts that 145 to 244 million DALYs would be averted across the six scenarios if vaccination occurred at age five. Most of these health benefits would occur in lower-middle-income countries (Fig. 1). This is due to the population size of those countries, their demographic structure, and the large burden of long-term debilitating diseases associated with Strep A (e.g., RHD) they face. If vaccination were administered at birth, the total number of DALYs averted would range from 76 to 153 million across the six scenarios. The difference in health benefits between child vaccination and infant vaccination is due mostly to the age distribution of the diseases associated with Strep A (i.e., children bear a larger burden than infants) and the assumed waning of vaccine effectiveness.

### Estimated global benefits of Strep A vaccination

**Fig. 2:**
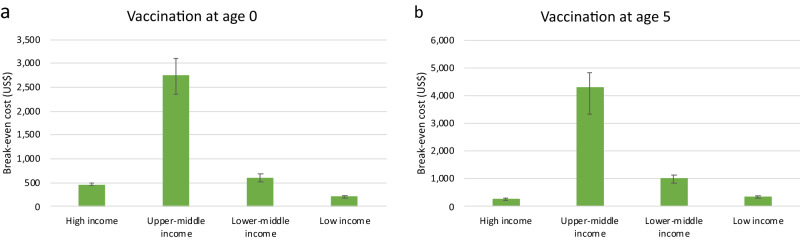
Average break-even cost across the six vaccination scenarios by income group (US$). Panel **a** shows the average break-even cost when vaccination occurs at age 0, and Panel **b** shows the average break-even cost when vaccination occurs at age 5. We assume a 3% discount rate and VSLY equal to three times GDP per capita (constant 2015 US$). GDP per-capita levels and growth rates differ by income group. The colored bars represent the average break-even cost across the six vaccination scenarios, while the black bars denote its variation. Please note that the scales of the y-axes differ between the two panels.

Assuming a 3% yearly discount rate and VSLY equal to three times gross-domestic-product (GDP) per capita, the largest overall benefits of Strep A vaccination would be experienced in upper-middle-income countries. Fig. 2 represents the maximum cost per vaccinated individual that would make Strep A vaccination economically beneficial. The break-even cost is significatively larger in upper-middle-income countries than in other income groups. For example, if vaccination were administered at age five, it would be an economically viable option in upper-middle-income countries as long as the average cost per vaccinated individual were less than $4300; in lower-middle-income countries, Strep A vaccination would pass a benefit-cost test if the cost per vaccinated individual were less than $1000. The higher break-even cost in upper-middle-income countries is due to a combination of relatively higher vaccine-preventable disease burden in comparison to high-income countries and relatively higher VSLY in comparison to lower-middle-income and low-income countries. Additionally, these results depend on the positive economic outlook in upper-middle income countries (i.e., relatively large projected income and economic growth).

**Fig. 3:**
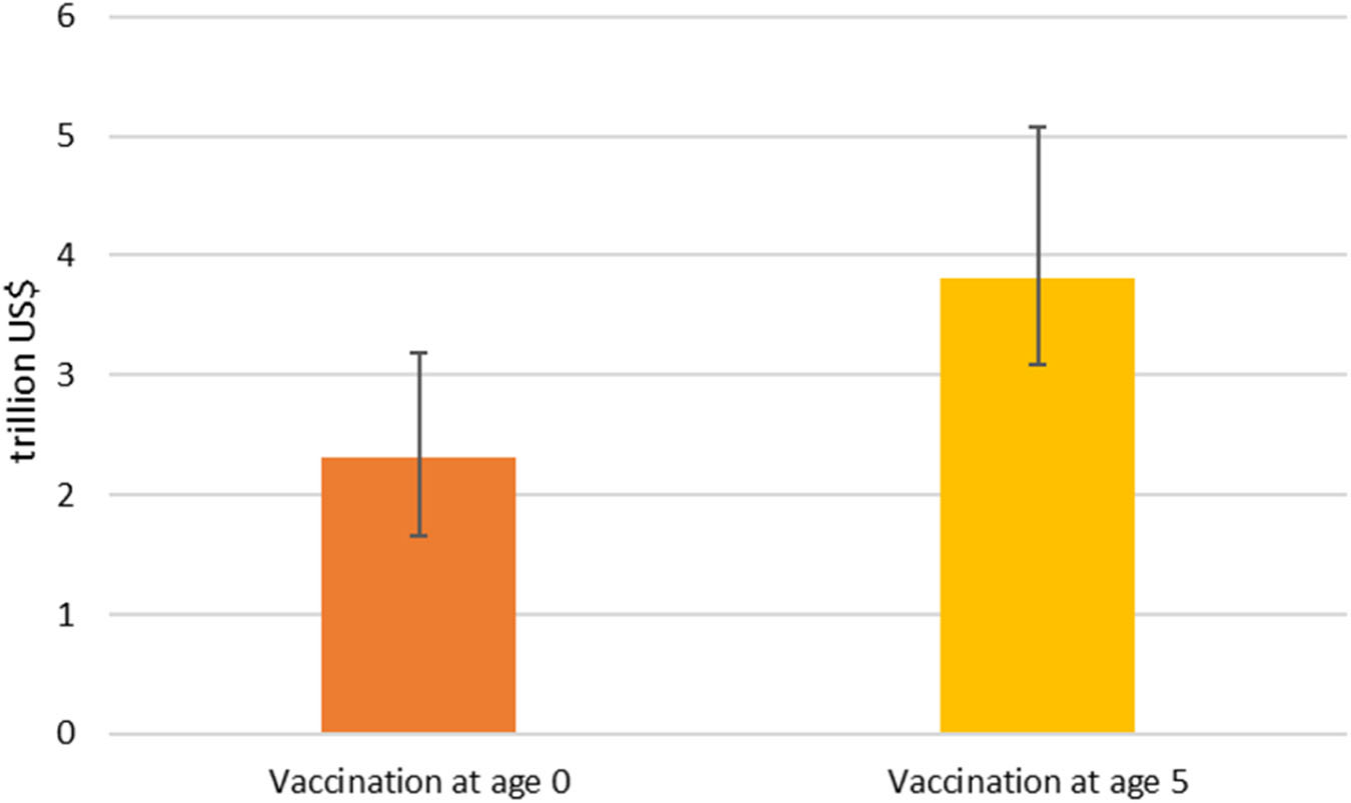
Average total benefits of Strep-A vaccination across the six vaccination scenarios from 2022 to 2051 (in trillions of US$). 3% discount rate and VSLY evaluated at three times global GDP per capita. Global GDP per capita is equal to $11,000 (constant 2015 US$). The colored columns represent the average benefits across the six scenarios, while the black bars represent the variation in benefits across the different scenarios.

Fig. 3 summarizes the average global benefits of prospective Strep A vaccination across the six vaccination scenarios. These benefits can be compared with the costs of developing, producing, and delivering vaccines to estimate the overall societal return on investing in Strep A vaccines. To avoid the undervaluation of benefits experienced by lower-income countries^17^, we adopt a single global estimate of VSLY to be applied to all countries when assessing the global benefits of Strep A vaccination. When the discount rate is equal to 3% and VSLY is equal to three times global GDP per capita, the average lifetime benefits for 30 birth cohorts across the six Strep A vaccination scenarios amount to $2.3 trillion if the vaccine were administered at birth ($1.7 to $3.2 trillion) and $3.8 trillion if the vaccine were administered at age five ($3.1 to $5.1 trillion). These figures are equivalent to 2.7% and 4.4%, respectively, of global income in 2021^41^, and they amount to 2,300 and 3,800 times the roughly $1 billion it has historically cost both to develop a successful vaccine (in risk-adjusted terms) and build a dedicated manufacturing facility for a single antigen^42^. These figures are also 57.5 and 95 times the high-end estimate of approximately $40 billion for the amount spent by the United States government directly on COVID-19 vaccine R&D and manufacturing^43^. While there will be additional recurring costs associated with manufacturing and delivery, investment in Strep A vaccination appears likely to yield a substantial societal return.

The chosen normative assumptions play a fundamental role in the estimation of the value of Strep A vaccination. With more favorable normative assumptions (discount rate of 1% and VSLY equal to five times global GDP per capita), the average benefits of Strep A vaccination increase to $6.96 trillion for infant vaccination and $11.46 trillion for childhood vaccination (respectively, 8.0% and 13.2% of 2021 global income) (Table 2). With normative assumptions that place less value on future benefits (5% discount rate) and on saving lives (VSLY equal to global GDP per capita), average benefits range from $460 billion for infant vaccination to $750 billion for childhood vaccination (respectively, 0.5% and 0.9% of 2021 global income).

**Table 2:** Benefits of Strep-A vaccination by scenario and normative assumptions (in trillions of US$).

	Infant vaccination			Childhood vaccination		
	Low value	Baseline	High value	Low value	Baseline	High value
Scenario 1	0.55	2.73	8.08	1.00	5.07	15.32
Scenario 2	0.61	3.19	9.92	0.97	5.08	15.80
Scenario 3	0.42	2.03	5.77	0.69	3.27	9.17
Scenario 4	0.47	2.36	7.06	0.67	3.26	9.44
Scenario 5	0.33	1.65	4.90	0.61	3.10	9.39
Scenario 6	0.37	1.93	6.02	0.59	3.09	9.66
Average	0.46	2.32	6.96	0.75	3.81	11.46

Baseline: 3% discount rate; VSLY is equal to three times global GDP per capita.

Low value: 5% discount rate; VSLY is equal to global GDP per capita.

High value: 1% discount rate; VSLY is equal to five times GDP per capita.

GDP per capita is equal to $11,000.

## Discussion

Vaccination confers a wide range of health, economic, and social benefits. Beyond direct prevention of morbidity and mortality and future healthcare costs, vaccination benefits that are likely to contribute substantially to Strep A vaccination’s societal value include reduction of antibiotic consumption and AMR; improved educational attainment, school attendance, and cognition among children; increased labor force participation, hours worked, and income among adults; and improved social equity both across and within populations. In monetary terms, we estimate that vaccinating children born in just the next 30 years against Strep A would be worth trillions of dollars to society globally. The cumulative cost of mid- to late-stage development, production, distribution, and delivery of Strep A vaccines is likely to come to a small fraction of this amount.

Our estimates of the global benefits of Strep A vaccination have several limitations. First, they do not fully account for non-internalized benefits of vaccination—i.e., any benefits that the vaccinated individual would not themselves enjoy and therefore not value. Examples of non-internalized benefits include population-wide AMR mitigation, reduced public health costs, indirect (herd) effects of vaccination, and potentially the positive impact of disease reduction on the mental health and quality of life of family or household members. In particular, the benefits accruing from reduced antibiotic consumption and AMR prevention are likely to be significant. Experience with other childhood vaccinations recently introduced into low- and middle-income countries suggests that substantial reduction in antibiotic prescribing and consumption is a realistic proposition. The addition of pneumococcal conjugate vaccines and live attenuated rotavirus vaccines to the WHO’s Expanded Programme on Immunization was estimated to result in 19.7% and 11.4% protection against antibiotic-treated episodes of acute respiratory infection and diarrhea, respectively, in young children in low- and middle-income countries^44^.

Second, since VSLY estimates for lower-income countries are lacking, we measure VSLY as a proportion of per-capita income. We measure income in terms of GDP at constant 2015 US$. Alternative measures of income include GDP based on purchasing power parity (PPP) exchange rates (rather than market exchange rates) and gross national income (GNI)^37,45^. The choice of income measure (GDP vs. GNI and PPP exchange rates vs. market exchange rates) will affect VSLY estimates and their relative difference across countries. Note, however, that the choice of income measure is likely to have only a minor impact on the estimated global societal benefits from Strep A vaccination due to the assumption of common VSLY estimates.

Third, due to the lack of studies on willingness to pay for Strep A incidence risk reduction, we proxy the value of averting a year lived with Strep A-related disability with VSLY. It is worth noting that our measure of the benefits derived from averting nonfatal cases of Strep A therefore reflects the value of averting a year lived with disability in general rather than willingness to pay specifically for Strep A risk reduction or Strep A vaccination. In addition, we implicitly assume that extending the length of life (possibly in poor health) is as valuable as increasing the quality of remaining life.

Fourth, the estimates do not fully account for distributional implications. To avoid the ethically questionable result that health benefits to high-income countries are valued more than equal health benefits to lower-income countries (due to the positive dependence of VSLY on income), we adopt a common VSLY for all countries. More sophisticated approaches would require the estimation of country-specific VSLY estimates and the adoption of a set of distribution weights to account for income differences across countries and for any ethical views about equity and distribution of net benefits from vaccination (e.g., health benefits to poor countries may be considered more morally valuable than equal benefits to higher-income countries).

Finally, we have only estimated Strep A vaccination’s benefits, which must be compared with its costs for a proper accounting of its value. Once costs are known, metrics such as benefit-cost ratio or return-on-investment can be used to determine the overall value of investing in the development, manufacture, and delivery of Strep A vaccines and compare that value to other potential uses of (limited) financial resources. For example, policymakers could compare the return-on-investment from Strep A vaccination with the return-on-investment from improved Strep A treatment.

Notwithstanding these limitations, the estimated magnitude of benefits from prospective Strep A vaccination appears to be large. In the baseline case, we estimate that the lifetime benefits of Strep A vaccination for 30 birth cohorts amount on average to 2.7% of global 2021 income if vaccination occurs at birth and 4.4% of global 2021 income if vaccination occurs at age five. Based on these findings, substantial investment in the development, manufacture, and delivery of Strep A vaccines is likely merited from a global societal perspective. However, progress on Strep A vaccine development has been relatively slow since the lifting in 2005 of a U.S. Food and Drug Administration moratorium that had inhibited research^46^. There are currently only eight Strep A vaccine candidates on a product development track, and none has yet advanced past Phase 1 clinical trials^6^. Considering the high estimated global societal benefits from safe and effective Strep A vaccination, the lack of forthcoming commercial investment into costly late-stage clinical development suggests that intervention may be warranted on the part of governments or global donors^47^.

Future research in this area may include generating more refined and comprehensive estimates of prospective Strep A vaccination’s societal benefits, perhaps through an integrated lifecycle model and social welfare function approach^48^. The costs of developing, manufacturing, and delivering Strep A vaccines globally should also be estimated to allow for comparison.

## Methods

### Epidemiological model

To quantify the global societal benefits of prospective Strep A vaccination, we adopt the results of a static cohort model developed by Giannini et al. (2023)^16^. The model was developed to project the country-specific number of episodes or cases of clinical disease, deaths, and DALYs averted by vaccination. The model considers five clinical diseases: cellulitis, impetigo, invasive disease, pharyngitis, and RHD. Deaths derive from invasive disease and RHD. Only the direct protective effects of vaccination are included (i.e., health impacts experienced by the vaccinated individuals). Indirect (herd) effects are excluded. The reduction in disease burden is in direct proportion to vaccine efficacy, vaccine coverage, and vaccine-derived immunity. The description of the model here is from Giannini et al. (2023)^16^. More details can be found in the cited paper.

The pre-vaccination disease burden is based on country- and age-specific incidence rates for cellulitis and RHD and global age-specific prevalence for impetigo from the 2019 Global Burden of Disease (GBD) study^49^. For pharyngitis and invasive disease, the pre-vaccination disease burden is based on global age-specific rates from systematic reviews conducted as part of the Strep A Vaccine Global Consortium (SAVAC) project. Mortality risk was limited to 28 days from hospitalization for invasive disease and to 10 years from disease onset for RHD. Country- and age-specific rates of Strep A burden were assumed to remain constant in the future. The model did not include acute post-streptococcal glomerulonephritis and acute rheumatic fever in the analysis due to data limitations on prevalent burden estimates.

Demographic estimates for the country-, year-, and age-specific population; all-cause mortality rates; and remaining life expectancy are based on the 2019 United Nations World Population Prospects^50^. The model uses non-sex-specific projected (2020 - 2100) interpolated age-specific (0 - 99 years) population and age-grouped (covering the same age range) all-cause mortality probabilities and remaining life expectancy estimates. Age groups for all-cause mortality and life expectancy are in 5-year bands, apart from 0 – 4 years (age 0 is in a separate group), assuming uniformity within groups for mortality and remaining life expectancy. Any projection of lifetime burden that went beyond 2100 assumed the same population, all-cause mortality, and remaining life expectancy values as for 2100. The country- and age-specific population numbers were used to estimate the population at age of vaccination, and then all-cause mortality probabilities were used to estimate the modelled population at each age over a cohort’s lifetime.

Disability weights used for the calculation of years lived with disability are from the GBD study^51^, and YLD (years lived with disability) were attributed to the years of prevalence. The durations for pharyngitis, impetigo, invasive disease, and cellulitis were estimated to be 5 days, 15.5 days, 10 days, and 16.4 days, respectively, based on the GBD-reported prevalence divided by incidence^49^. The duration for RHD was assumed to be the remaining life expectancy from the onset of the condition.

Vaccination occurs either at birth or at age five. The vaccine efficacy assumptions are based on the WHO-preferred product characteristics for Strep A vaccines^5^. These include 80% efficacy against pharyngitis and impetigo, 70% efficacy against invasive disease and cellulitis, and 50% efficacy against RHD.

The model considers six vaccination scenarios that differ in terms of years of vaccine introduction, coverage, and waning dynamics. Table 3 summarizes the assumptions underlying the vaccination scenarios.

**Table 3:** Vaccination scenarios: Potential vaccination scenarios with varying years of vaccine introduction, maximum coverage, and vaccine-derived immunity dynamics.

Scenario	Year of vaccine introduction	Maximum coverage	Durability of vaccine-derived immunity
1	Country-specific (2022–2034)	Country-specific (9–99%)	Full efficacy for 10 years
2	Country-specific (2022–2034)	Country-specific (9–99%)	Linear waning over 20 years
3	2022	50%	Full efficacy for 10 years
4	2022	50%	Linear waning over 20 years
5	Country-specific (2022–2034)	50%	Full efficacy for 10 years
6	Country-specific (2022–2034)	50%	Linear waning over 20 years

The waning dynamics of vaccine-derived immunity were modelled in two ways: (i) vaccine-induced immune protection at maximum efficacy for 10 years and null thereafter and (ii) waning linearly with an annual reduction in efficacy equivalent to 5% of maximum efficacy for 20 years and null thereafter (i.e., waning to 50% of maximum efficacy after 10 years). The year of vaccine introduction was assumed to be 2022 or country-specific ranging from 2022 to 2034, with initial coverage at 10% of maximum coverage. The vaccine coverage was assumed to scale up linearly during the first 10 years after introduction to reach either a maximum of 50% coverage for all countries or a country-specific coverage ranging from 9 to 99%. Country-specific coverage values and year of introduction are based on past trends for Hib3 or the third dose of diphtheria, tetanus, and pertussis vaccine where Hib3 values were unavailable.

In estimating the global societal benefits of prospective Strep A vaccination, we consider the lifetime health impacts for 30 vaccinated cohorts, from 2022 to 2051 (i.e., either birth cohorts from 2022 to 2051, or cohorts of individuals that reach age five from 2022 to 2051). For countries that introduce the vaccine after the year 2022 (in scenarios 1–2 and 5–6), we consider the lifetime health benefits experienced by the birth/age-five cohorts from the year of vaccine introduction to 2051. We consider health impacts in terms of DALYs. The epidemiological model provides country- and age-specific numbers of DALYs averted for each vaccination scenario and age of vaccination (birth or age five).

### Estimation of the global societal benefits of Strep A vaccination

To determine the socioeconomic benefits associated with the number of DALYs averted, we rely on the concepts of value-per-statistical-life-year (VSLY) and value-per-statistical-disability (VSD). VSLY is the marginal rate of substitution between income and life expectancy^52^, and it is derived from individuals’ willingness to trade off small changes in income for small changes in mortality risk. VSD is the marginal rate of substitution between income and health-related quality of life, and it is derived from individuals’ willingness to trade off small changes in income for small changes in nonfatal morbidity risk. VSLY depends on the value placed by individuals on extending their life expectancy by an additional year. VSD depends on the value placed by individuals on living a year in good health rather than in a disability state. Conceptually, VSLY and VSD include both the intrinsic and instrumental values of living longer and in good health. For these reasons, the VSLY/VSD concepts are well-placed to capture (at least part of) the broad benefits of vaccination.

VSLY estimates are typically obtained by dividing the population-average value-per-statistical-life (VSL) by the average remaining life expectancy. In turn, VSL is derived from the rate at which individuals are willing to trade off small changes in income for small changes in risk of death. For example, if individuals in a group of 1000 people are each willing to pay $1000 to reduce their risk of death by 0.1%, the value per statistical life in this group is equal to $1,000,000. This does not mean that each individual would pay $1,000,000 to guarantee their own survival. Rather, it means that each would agree to pay an equal share of $1,000,000 (i.e., $1000) to fund a project that reduces the expected number of fatalities in the group by one. VSL estimates are based on individuals’ reported preferences or on individuals’ consumption and work behaviors, and they typically vary by income, baseline risk, and age. In particular, VSL (and thus VSLY) is typically found to be increasing with income.

Empirical data on VSLY are lacking for lower-income countries. We follow standard guidelines for the conduct of benefit-cost analysis in the absence of scenario-specific willingness-to-pay estimates^37^ and assume that: i) VSLY (and VSL) is proportional to income and ii) the monetary value of benefits experienced by future generations is discounted at a constant yearly rate $r$. Estimates of VSLY are typically between one and five times per-capita income. In the simulation exercises, we vary the yearly discount rate $r$ from 1 to 5%. Because of lack of data on VSD estimates, we again follow the relevant literature and assume that the value of preventing one year of life with disability is also equal to VSLY.

For each country $i$, the full benefits ${B}_{i}$ of Strep A vaccination for the thirty cohorts under analysis are defined as the present discounted value of future monetary benefits:

1 $${B}_{i}=\mathop{\sum }\limits_{t=0}^{T}{DAL}{Y}_{{it}}\frac{{VSL}{Y}_{{it}}}{{\left(1+r\right)}^{t}}$$

In the previous formula, $t=0$ represents the year 2022, and $t=T$ represents the maximum length of life of individuals who are vaccinated in the year 2051. For example, $T=2151$ if vaccination occurs at birth and individuals are expected to live at most 100 years. ${DAL}{Y}_{{it}}$ is the overall number of DALYs averted in country $i$ and period $t$. Note that if the first year of vaccine introduction in a country is after 2022, ${DAL}{Y}_{{it}}=0$ between 2022 and the year before vaccine introduction. ${VSL}{Y}_{{it}}$ is the value-per-statistical-life-year in country $i$ and period $t$, and $r$ is the constant yearly discount rate. In turn, VSLY is equal to:

2 $${VSL}{Y}_{{it}}=\alpha {Y}_{{it}}=\alpha {Y}_{i0}{\left(1+{g}_{i}\right)}^{t}$$

where $\alpha$ is the degree of proportionality between VSLY and income (assumed to range from one to five), and ${Y}_{{it}}$ is the per-capita income in country $i$ and period $t$. Income is assumed to increase over time at the country-specific yearly rate ${g}_{i}$.

**Fig. 4:**
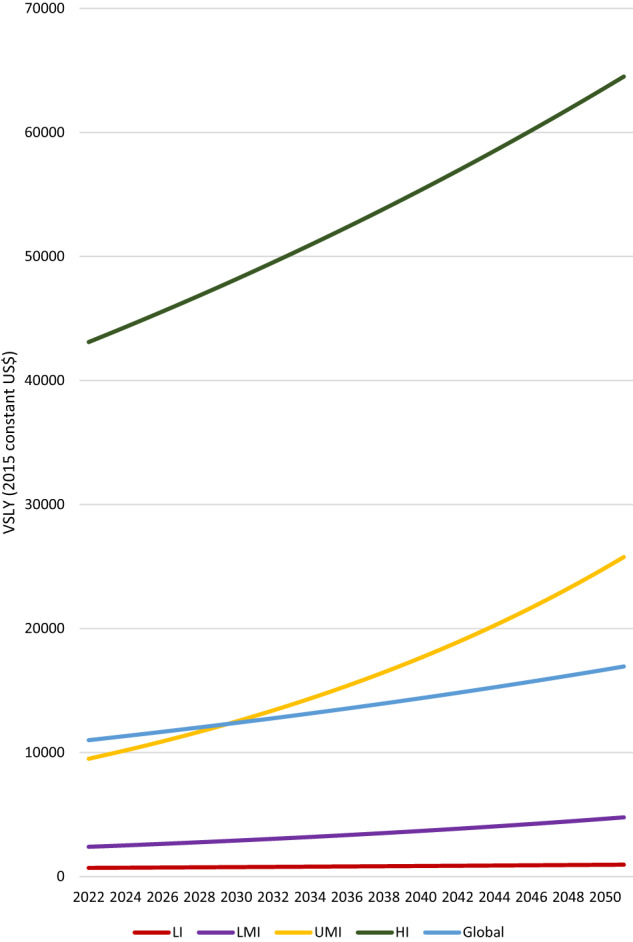
Trajectories of value-per-statistical-life-year (VSLY) by country-income group (2015 US$). VSLY is assumed to be equal to GDP per capita. Each line represents the trajectory of VSLY over time (from 2022 to 2051) by country-income group and for the whole world. Data on per-capita GDP at the beginning of the simulation and per-capita annual GDP growth are reported in Table 4.

To measure income in the initial year, we use World Bank data on per-capita gross domestic product (GDP) in 2019 (at 2015 constant US$), i.e., before the arrival of the COVID-19 pandemic. Per-capita GDP growth rates are assumed to be equal to the rates experienced in 2019. GDP levels and GDP growth data by country-income group are displayed in Table 4. As an example, Fig. 4 depicts the estimated annual VSLY under the assumption that VSLY is equal to GDP per capita (i.e., $$\alpha =1$$). These values have been computed by plugging the figures provided in Table 4 into Eq. (2).

**Table 4:** Assumptions about per-capita GDP and economic growth.

Country group	Per-capita GDP at the beginning of the simulation (2015 constant US$)	Per-capita annual GDP growth
Low income	$700	1.1%
Lower-middle income	$2400	2.4%
Upper-middle income	$9500	3.5%
High income	$43,100	1.4%
Global	$11,000	1.5%

To compute the country-specific break-even point ${c}_{i}$, i.e., the maximum cost per vaccinated person that would make Strep A vaccines economically viable, we solve the following expression:

3 $${B}_{i}-{c}_{i}\mathop{\sum }\limits_{t=0}^{\bar{T}}\frac{{N}_{{it}}}{{\left(1+r\right)}^{t}}=0$$

where $\bar{T}$ is the last cohort vaccinated (2051 birth cohort or cohort at age five in 2051), and ${N}_{{it}}$ is the number of individuals vaccinated in country $i$ and period $t$. The number of vaccinated individuals depends on the vaccination scenario, i.e., the year of vaccine introduction and coverage.

The dependence of VSLY on income can have unacceptable ethical implications. In particular, since a well-off individual may be willing to pay a larger amount of money than a less well-off individual for the same change in risk of death, the use of country-specific VSLY estimates implies that the lives and interests of the well-off count more than those of the less well-off^17^. To avoid undervaluation of benefits experienced by lower-income countries, in the computation of the global benefits of Strep A vaccination, we adopt a single global estimate of VSLY to be applied to all countries and assume that it is equal to one to five times global GDP per-capita.

### Reporting summary

Further information on research design is available in the Nature Research Reporting Summary linked to this article.

### Supplementary information

Reporting Summary

## Data Availability

The data underlying the analyses presented in this paper are publicly available from the following sources: Vos, T. et al. Global burden of 369 diseases and injuries in 204 countries and territories, 1990–2019: a systematic analysis for the Global Burden of Disease Study 2019. *Lancet* **396**, 1204–1222 (2020). 10.1016/S0140-6736(20)30925-9. UNDP. World Population Prospects 2019. (2019). https://population.un.org/wpp/. Salomon, J. A. et al. Disability weights for the Global Burden of Disease 2013 study. *Lancet. Glob. Heal*. **3**, e712–23 (2015). 10.1016/S2214-109X(15)00069-8. The World Bank. World Development Indicators. (2022). https://databank.worldbank.org/source/world-development-indicators.

## Code availability

The program code and data for the epidemiological model is available as an R package, *GASImpactModel* (https://github.com/fionagi/GASImpactModel), and modeling analysis can be conducted through the R Shiny web application (https://github.com/fionagi/GASImpactModel_App).
