# Supplementary material for: The full health, economic, and social benefits of prospective Strep A vaccination
Source: NPJ Vaccines. 2023 Oct 30;8:166. doi: 10.1038/s41541-023-00758-z (PMC10616198; doi:10.1038/s41541-023-00758-z)
Supplement: Supplementary file 1 — Reporting Summary [file 41541_2023_758_MOESM1_ESM.pdf]

nature portfolio

Corresponding author(s): Daniel Cadarette

Last updated by author(s): Mar 29, 2023

Reporting Summary

Nature Portfolio wishes to improve the reproducibility of the work that we publish. This form provides structure for consistency and transparency in reporting. For further information on Nature Portfolio policies, see our Editorial Policies and the Editorial Policy Checklist.

Statistics

For all statistical analyses, confirm that the following items are present in the figure legend, table legend, main text, or Methods section.

n/a	Confirmed	
☒	☐	The exact sample size (n) for each experimental group/condition, given as a discrete number and unit of measurement
☒	☐	A statement on whether measurements were taken from distinct samples or whether the same sample was measured repeatedly
☒	☐	The statistical test(s) used AND whether they are one- or two-sided<br>*Only common tests should be described solely by name; describe more complex techniques in the Methods section.*
☒	☐	A description of all covariates tested
☒	☐	A description of any assumptions or corrections, such as tests of normality and adjustment for multiple comparisons
☒	☐	A full description of the statistical parameters including central tendency (e.g. means) or other basic estimates (e.g. regression coefficient) AND variation (e.g. standard deviation) or associated estimates of uncertainty (e.g. confidence intervals)
☒	☐	For null hypothesis testing, the test statistic (e.g. F, t, r) with confidence intervals, effect sizes, degrees of freedom and P value noted<br>*Give P values as exact values whenever suitable.*
☒	☐	For Bayesian analysis, information on the choice of priors and Markov chain Monte Carlo settings
☒	☐	For hierarchical and complex designs, identification of the appropriate level for tests and full reporting of outcomes
☒	☐	Estimates of effect sizes (e.g. Cohen's d, Pearson's r), indicating how they were calculated

Our web collection on statistics for biologists contains articles on many of the points above.

Software and code

Policy information about availability of computer code

Data collection	*Provide a description of all commercial, open source and custom code used to collect the data in this study, specifying the version used OR state that no software was used.*
Data analysis	The epidemiological model was developed using the R statistical software. The program code and data for the epidemiological model are available at https://github.com/fionagi/GASImpactModel

For manuscripts utilizing custom algorithms or software that are central to the research but not yet described in published literature, software must be made available to editors and reviewers. We strongly encourage code deposition in a community repository (e.g. GitHub). See the Nature Portfolio guidelines for submitting code & software for further information.

Data

Policy information about availability of data

All manuscripts must include a data availability statement. This statement should provide the following information, where applicable:

- Accession codes, unique identifiers, or web links for publicly available datasets
- A description of any restrictions on data availability
- For clinical datasets or third party data, please ensure that the statement adheres to our policy

The data underlying the analyses presented in this paper are publicly available from the following sources:

Vos, T. et al. Global burden of 369 diseases and injuries in 204 countries and territories, 1990-2019: a systematic analysis for the Global Burden of Disease Study 2019. Lancet 396, 1204–1222 (2020). DOI: 10.1016/S0140-6736(20)30925-9

UNDP. World Population Prospects 2019. (2019). https://population.un.org/wpp/

Salomon, J. A. et al. Disability weights for the Global Burden of Disease 2013 study. Lancet. Glob. Heal. 3, e712-23 (2015). DOI: 10.1016/S2214-109X(15)00069-8

The World Bank. World Development Indicators. (2022). https://databank.worldbank.org/source/world-development-indicators

Human research participants

Policy information about studies involving human research participants and Sex and Gender in Research.

Reporting on sex and gender	Not applicable
Population characteristics	Not applicable
Recruitment	Not applicable
Ethics oversight	Not applicable

Note that full information on the approval of the study protocol must also be provided in the manuscript.

Field-specific reporting

Please select the one below that is the best fit for your research. If you are not sure, read the appropriate sections before making your selection.

☐ Life sciences ☒ Behavioural & social sciences ☐ Ecological, evolutionary & environmental sciences

For a reference copy of the document with all sections, see nature.com/documents/nr-reporting-summary-flat.pdf

Behavioural & social sciences study design

All studies must disclose on these points even when the disclosure is negative.

Study description

This is a mixed-methods study incorporating qualitative analysis based on targeted literature review and expert consultation and quantitative analysis of publicly available data.

Research sample

This study involved existing datasets of population-level data.

he data underlying the analyses presented in this paper are publicly available from the following sources:

Vos, T. et al. Global burden of 369 diseases and injuries in 204 countries and territories, 1990-2019: a systematic analysis for the Global Burden of Disease Study 2019. Lancet 396, 1204–1222 (2020). DOI: 10.1016/S0140-6736(20)30925-9

UNDP. World Population Prospects 2019. (2019). https://population.un.org/wpp/

Salomon, J. A. et al. Disability weights for the Global Burden of Disease 2013 study. Lancet. Glob. Heal. 3, e712-23 (2015). DOI: 10.1016/S2214-109X(15)00069-8

The World Bank. World Development Indicators. (2022). https://databank.worldbank.org/source/world-development-indicators

Sampling strategy	Not applicable
Data collection	No new data were collected for this study.
Timing	This study involved existing datasets of population-level data. GBD data and UNDP data are from 2019. Disability weights for GBD data are from 2013. World Bank data are from 2022.
Data exclusions	No data were excluded from the analyses.
Non-participation	Not applicable
Randomization	Not applicable

Reporting for specific materials, systems and methods

We require information from authors about some types of materials, experimental systems and methods used in many studies. Here, indicate whether each material, system or method listed is relevant to your study. If you are not sure if a list item applies to your research, read the appropriate section before selecting a response.

Materials & experimental systems

n/a	Involved in the study
☒	☐ Antibodies
☒	☐ Eukaryotic cell lines
☒	☐ Palaeontology and archaeology
☒	☐ Animals and other organisms
☒	☐ Clinical data
☒	☐ Dual use research of concern

Methods

n/a	Involved in the study
☒	☐ ChIP-seq
☒	☐ Flow cytometry
☒	☐ MRI-based neuroimaging
